# Circulation of Ngari Virus in Livestock, Kenya

**DOI:** 10.1128/msphere.00416-22

**Published:** 2022-12-06

**Authors:** Dorcus C. A. Omoga, David P. Tchouassi, Marietjie Venter, Edwin O. Ogola, Georg Joachim Eibner, Anne Kopp, Inga Slothouwer, Baldwyn Torto, Sandra Junglen, Rosemary Sang

**Affiliations:** a International Centre of Insect Physiology and Ecology, Nairobi, Kenya; b Zoonotic, Arbo, and Respiratory Virus Research Program, Centre for Viral Zoonoses, Department of Medical Virology, Faculty of Health, University of Pretoria, Gezina, South Africa; c Institute of Virology, Charité Universitätsmedizin Berlin, Corporate Member of Free University Berlin, Humboldt-University Berlin, and Berlin Institute of Health, Berlin, Germany; d Centre for Virus Research, Kenya Medical Research Institute, Nairobi, Kenya; U.S. Centers for Disease Control and Prevention

**Keywords:** Ngari virus, orthobunyavirus, hemorrhagic fever, livestock, Kenya

## Abstract

Ngari virus (NRIV) is a mosquito-borne reassortant orthobunyavirus that causes severe febrile illness and hemorrhagic fever in humans and small ruminants. Due to limited diagnostics and surveillance, NRIV has only been detected sporadically during Rift Valley fever virus outbreaks. Little is known on its interepidemic maintenance and geographic distribution. In this study, sera from cattle, goats, and sheep were collected through a cross-sectional survey after the rainy seasons between 2020 and 2021 in two pastoralist-dominated semiarid ecosystems, Baringo and Kajiado counties in Kenya. NRIV was detected in 11 apparently healthy animals (11/2,039, 0.54%) by RT-PCR and isolated in cell culture from seven individuals. Growth analyses displayed efficient replication in cells from sheep and humans in contrast to weak replication in goat cells. NRIV infection of a wide variety of different vector cells showed only rapid replication in Aedes albopictus cells but not in cells derived from other mosquito species or sandflies. Phylogenetic analyses of complete-coding sequences of L, M, and S segments of four viruses showed that the Kenyan sequences established a monophyletic clade most closely related to a NRIV sequence from a small ruminant from Mauritania. NRIV neutralizing reactivity in cattle, goats, and sheep were 41.6% (95% CI = 30 to 54.3), 52.4% (95% CI = 37.7 to 66.6), and 19% (95% CI = 9.7 to 33.6), respectively. This is the first detection of NRIV in livestock in Kenya. Our results demonstrate active and undetected circulation of NRIV in the three most common livestock species highlighting the need for an active one-health surveillance of host networks, including humans, livestock, and vectors.

**IMPORTANCE** Surveillance of vectors and hosts for infection with zoonotic arthropod-borne viruses is important for early detection and intervention measures to prevent outbreaks. Here, we report the undetected circulation of Ngari virus (NRIV) in apparently healthy cattle, sheep, and goats in Kenya. NRIV is associated with outbreaks of hemorrhagic fever in humans and small ruminants. We demonstrate the isolation of infectious virus from several animals as well as presence of neutralizing antibodies in 38% of the tested animals. Our data indicate active virus circulation and endemicity likely having important implications for human and animal health.

## INTRODUCTION

Ngari virus (NRIV) belongs to the *Bunyamwera orthobunyavirus* species (genus *Orthobunyavirus*, family *Peribunyaviridae*, order *Bunyavirales*) and is the only known naturally occurring reassortant in the Bunyamwera serogroup (1). The *Bunyamwera orthobunyavirus* species comprises 10 viruses: for example, Bunyamwera virus (BUNV), Germiston virus (GERV), and Shokwe virus (SHOV) (1–4). The tripartite genome of orthobunyaviruses contains linear, single-stranded, negative-sense RNA. The three segments are named small (S), medium (M), and large (L) according to their length. The S segment encodes the nucleocapsid (N) protein and the NSs nonstructural protein, the M segment encodes the two glycoproteins Gn and Gc and a nonstructural protein NSm, and the L segment, the RNA-dependent RNA-polymerase (5–8).

NRIV is a reassortant of BUNV (*Bunyamwera orthobunyavirus* species) and Batai virus (BATV, *Batai orthobunyavirus* species) (1, 6, 7) containing the L and S segments from BUNV and the M segment from BATV (6, 7, 9). Genetic reassortment among orthobunyaviruses has occasionally been reported and is considered the main evolutionary force leading to the emergence of new strains and species (3, 4, 10–13). Whereas BATV and BUNV cause mild symptoms, such as fever, joint pain, and rash in humans, NRIV is more virulent and can induce severe and fatal hemorrhagic fever (3, 5, 14–16). However, BATV can cause a more severe form of disease in livestock resulting in genetic defects and abortions (3, 17).

NRIV was first isolated from *Aedes simpsoni* mosquitoes in 1979 in South Eastern Senegal (5, 11, 18). It was later recovered from *Aedes* spp., *Culex* spp., and *Anopheles* spp. in Senegal, Burkina Faso, Central African Republic, and Madagascar (19). NRIV has also been detected in engorged ixodid ticks (Amblyomma variegatum, *Rhipicephalus geigyi*, and *Rh. [Boophilus]* spp.) collected from cattle in Guinea; however, there is no evidence that ticks can transmit the virus (20). The virus was first associated with human disease following isolation from two patients in Senegal in 1973 (5, 18), and subsequently implicated as a cause of hemorrhagic fever during a large hemorrhagic fever outbreak in North Eastern Kenya and southern Somalia in 1997 to 1998 (6, 7, 9). In a later study conducted between 2009 and 2012 in North Eastern Kenya, presence of NRIV-neutralizing antibodies was reported among febrile patients (21), indicating circulation of an understudied virus. With regard to animal infections, NRIV has only been isolated from small ruminants in Mauritania during the 2010 and 2015 Rift Valley Fever (RVF) outbreaks (14, 16, 22), both confirming cocirculation with rift valley fever virus (RVFV) and other orthobunyaviruses, BATV and BUNV.

BUNV and NRIV have mainly been identified in Africa. BUNV is considered endemic in certain African countries, including Rwanda, Kenya, Nigeria, Senegal, Uganda, Tanzania, Mozambique, Guinea, South Africa, DRC, and Madagascar (3, 14–16). BUNV has also been reported in Mexico and Argentina in birds and mosquitoes, and more recently as a cause of neurological disease and abortion in horses (20, 21, 23). BATV is distributed worldwide though commonly found in Asia and Europe (5, 20, 21, 23). Although the geographic range of these viruses suggests a restricted distribution, there is potential for spread due to globalization, human and animal movement, and environmental changes due to global warming.

NRIV has been found to circulate concurrently with RVFV during outbreaks and has been clinically misdiagnosed as RVF as the case during the 1997/1998 RVF outbreak in Kenya and Somalia and as malaria in Sudan (6, 18). The misdiagnosis was partly due to similarities in symptom presentation. Thus, its distribution and associated health impact could be grossly underestimated due to paucity of active surveillance, poor disease reporting systems, and lack of appropriate diagnosis.

In Kenya, since its initial isolation in humans, several studies have isolated or detected NRIV in different mosquito species, like Anopheles funestus (Tana Delta), *Aedes mcintoshi* (Garissa), and different tick species, like *Amblyomma gemma* (Garissa) and *Rhipicephalus pulchellus* (Isiolo) (8, 19, 24–26). Diverse tick and mosquito species have been found to be competent vectors for the virus in laboratory experiments (27, 28). However, there have not been any reports of NRIV circulation in any livestock species outside Mauritania.

Against the backdrop of poor surveillance, this study was initiated to improve our understanding of the circulation of NRIV in selected predominantly pastoral ecosystems in Kenya. We aimed to detect and characterize NRIV in serum samples collected from a network of livestock hosts, such as goat, sheep, and cattle in dryland ecosystems in Kenya. Findings of the study have implications for the sources of NRIV outbreaks in humans and for surveillance and control of NRIV.

## RESULTS

### Ngari virus infects sheep, goat, and cattle in Kenya.

**FIG 1:**
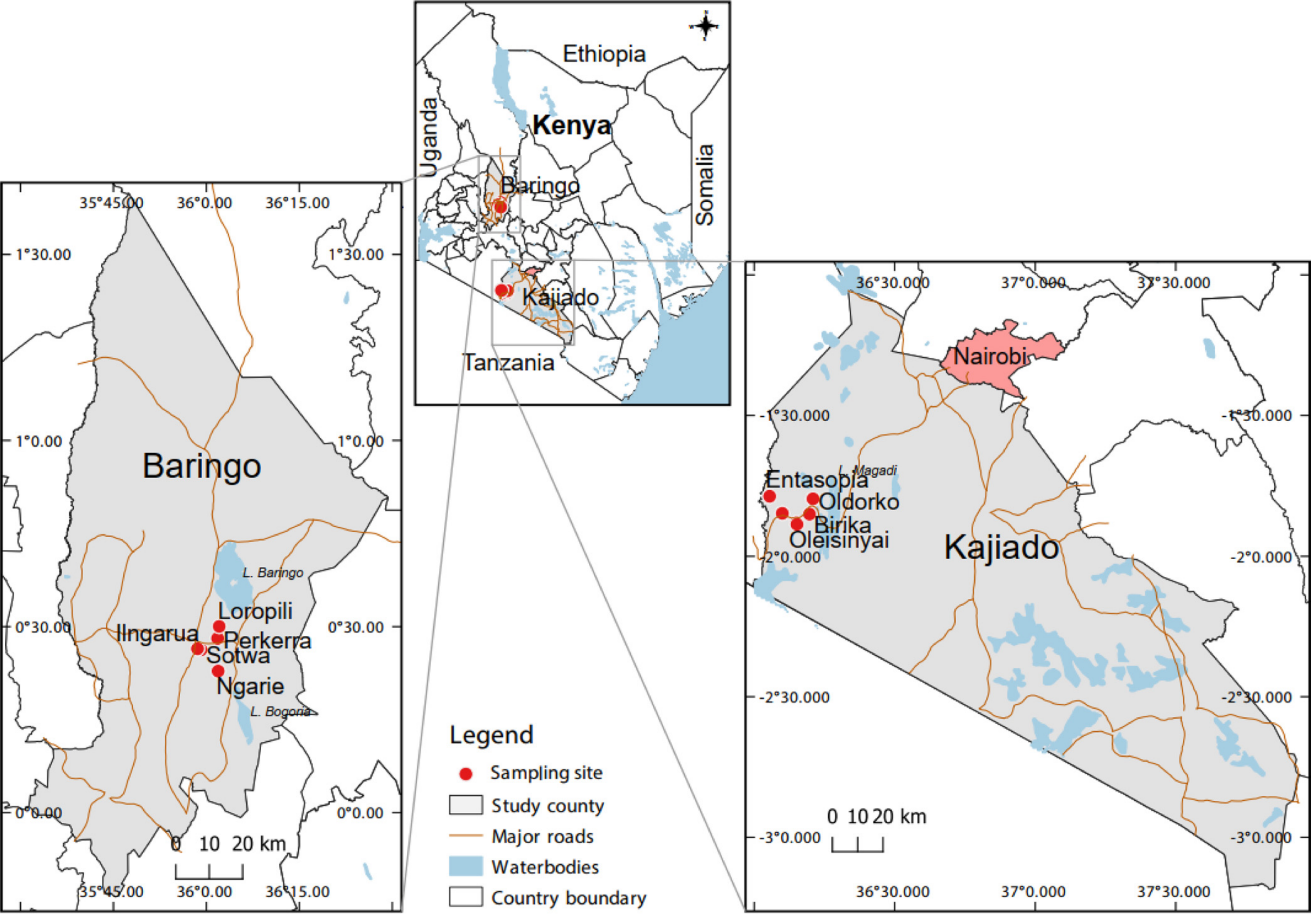
A map of Kenya showing the study sites in Baringo and Kajiado counties, respectively, shaded in gray. The red spots represent the selected locations within the counties where sampling was conducted. The maps were created in the open source GIS software, QGIS 3.22 using GPS coordinates and shape files derived from Natural Earth (http://www.naturalearthdata.com/, a free GIS data source) and Africa Open data (https://africaopendata.org/dataset/kenya-counties-shapefile, license Creative Commons) (43).

A total of 2,039 sera from apparently healthy cattle (*n* = 715), goats (*n* = 680), and sheep (*n* = 644) aged 1 to 3 years were sampled from selected sites inhabited by pastoral communities of Baringo and Kajiado counties in Kenya’s Rift Valley (Fig. 1). In total, 1,239 samples (60.8%) originated from Baringo county (cattle, *n* = 415 samples, goats, *n* = 420 samples, and sheep, *n* = 404 samples) and 800 samples (39.2%) originated from Kajiado county (cattle, *n* = 300 samples, goats, *n* = 260 samples, and sheep, *n* = 240 samples). NRIV was detected in 11 individual samples 11/2,039, 0.54% (95% CI = 0.29 to 0.98): three sera from cattle (3/715, 0.42%), three sera from goats (3/680, 0.44%), and four sera from sheep (4/644, 0.62/%) by RT-PCR of which two goat sera were from Kajiado county and the other nine from Baringo county (Table 1). None of the 11 NRIV positive samples were tested positive for any other arbovirus, including RVFV and BUNV.

**TABLE 1:** Origin of NRIV positive livestock samples

Sample-ID	Livestock species	Age (yrs)	Sex	Place of origin (county/location)	Dates of sample collection
KE-O52	Sheep	2	Female	Baringo (Sotwa)	9/22/2020
KE-O56	Sheep	2	Female	Baringo (Sotwa)	9/22/2020
KE-O93	Sheep	2	Male	Baringo (Loropilli)	9/23/2020
KE-O288	Sheep	2	Female	Baringo (Elketeiyo Sintaan)	5/19/2021
KE-C166	Goat	2	Male	Baringo (Lokuru)	9/26/2020
KE-C177	Goat	2	Male	Baringo (Lokuru)	9/27/2020
KE-C106	Goat	1	Female	Kajiado (Oldorko)	10/13/2020
KE-C174	Goat	2	Male	Kajiado (Entasopia)	10/15/2020
KE-B02	Cattle	1	Male	Baringo (Ilngarua)	9/16/2020
KE-B29	Cattle	2	Male	Baringo (Perkerra)	9/17/2020
KE-B35	Cattle	2	Male	Baringo (Perkerra)	9/17/2020

### NRIV tropism appears restrictive to *Aedes* cells while vertebrate cells are broadly susceptible.

We next attempted to isolate the virus from the 11 PCR-positive serum samples in cell culture. Seven samples from Baringo county consistently displayed similar cytopathic effects (CPE) 2 to 4 days postinoculation (dpi). Sequence analysis of extracted RNA from the supernatant of the isolates (goat: KE-C166, KE-C177; sheep: KE-O93 and KE-O288; cattle: KE-B02, KE-B29, and KE-B35) confirmed the isolation of NRIV in cell culture.

**FIG 2:**
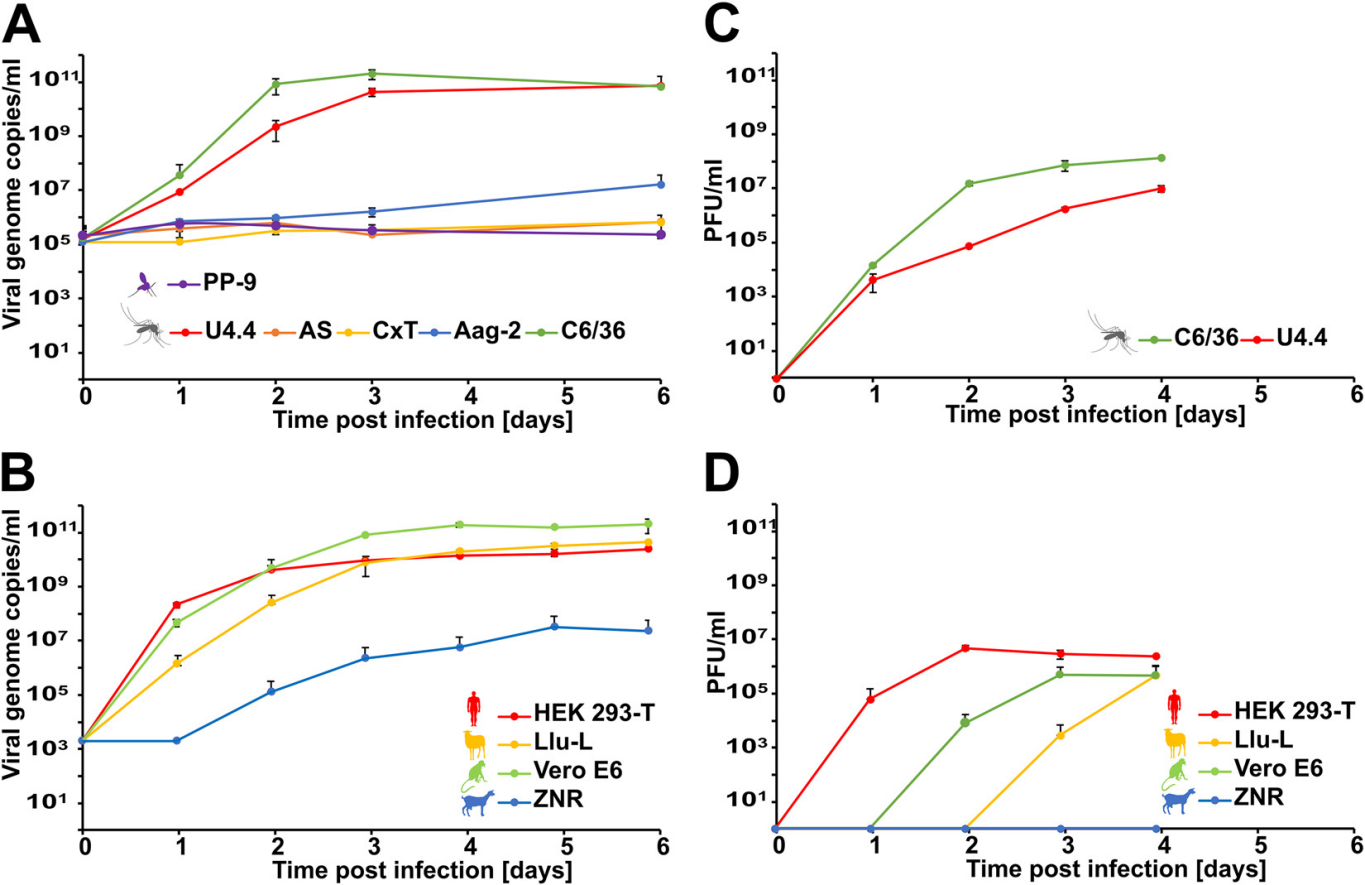
*In vitro* growth kinetics of NRIV in different cell lines. (A and C) Insect cell lines (U4.4, AS, CxT, Aag-2, C6/36, mosquito; and PP-9, sand fly) were infected with NRIV in duplicates with a multiplicity of infection (MOI) of 0.1. (B and D) Vertebrate cell lines (HEK293-T, human; Llu-L, sheep; VeroE6, primate; ZN-R, goat) were infected with NRIV in duplicates with a MOI of 0.01. Infectious cell culture supernatants were collected at the indicated time points for RNA extraction and virus titration. Virus concentrations are given in viral genome copies/mL (A and B) and PFU/mL (C and D).

Although NRIV has been detected in various mosquito species from different genera, *in vitro* growth analyses showed that NRIV replicated only in cell lines derived from Aedes albopictus (C6/36 and U4.4) but not in cells derived from *Anopheles* or *Culex* mosquitoes nor *Phlebotomus* sandflies (Fig. 2A) suggesting that *Aedes* derived cell lines are more susceptible for NRIV infection. In contrast, vertebrate cells were broadly susceptible for NRIV infection with peak genome copy numbers in cells derived from human, sheep, and nonhuman primates, and 1,000- to 10,000-fold lower replication rates in goat cells (Fig. 2B). Production of infectious virus particles correlated well with viral genome copy numbers in cell culture supernatants (Fig. 2C and D).

### The Kenyan NRIV isolates are most closely related to NRIV isolates from Mauritania.

**FIG 3:**
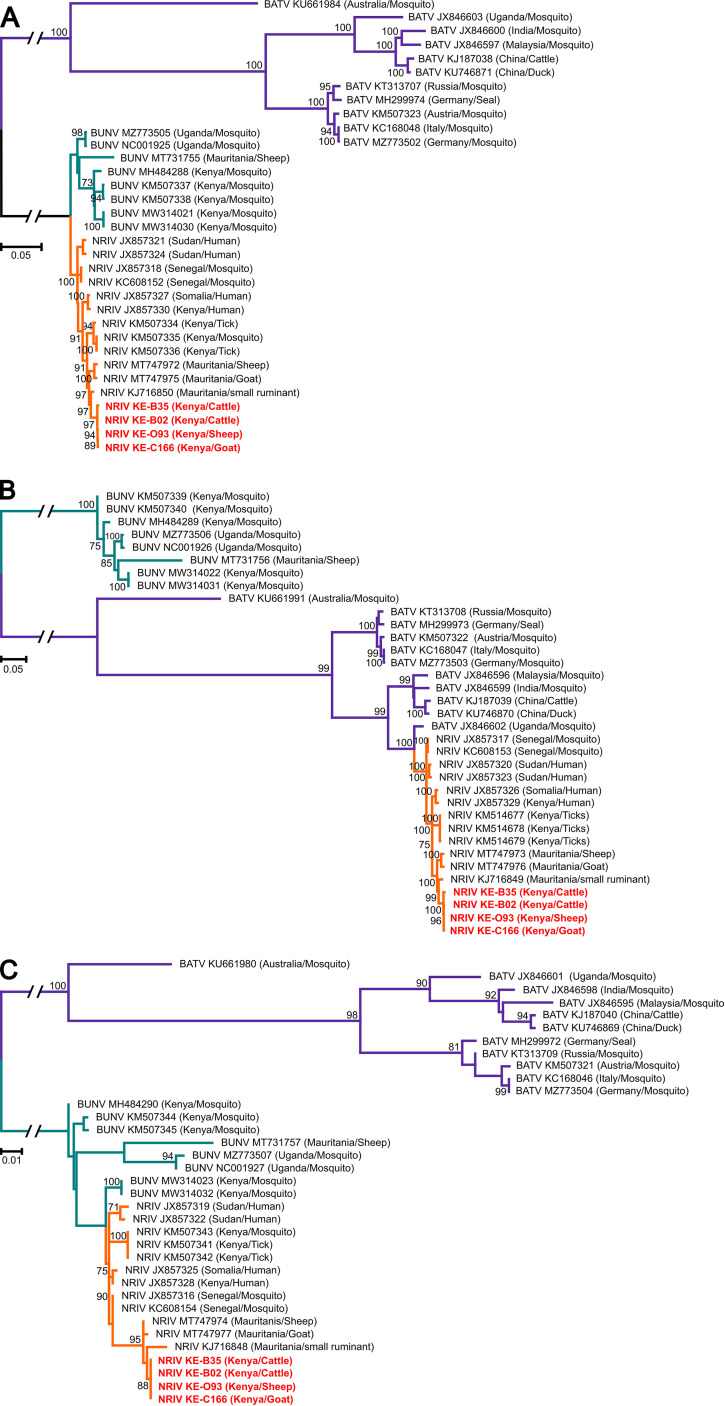
Phylogenetic analysis of the isolated NRIV in livestock from Baringo County based on the cds nucleotide sequences of the: (A) L segment (6,717 nt), (B) M segment (4,305 nt), and (C) S segment (702 nt) compared with other orthobunyaviruses. The samples in this study are highlighted in red. The sequences were aligned using MAFFT with E-insi and 100PAM. The ML tree was calculated using IQtree via CIPRES (Geneious plug-in at www.phylo.org) and nonparametric bootstrapping was used.

Entire NRIV genomes were sequenced from four virus isolates derived from sheep (KE-093), goat (KE-C166), and cattle (KE-B02 and KE-B35). All four genomes showed similar length for the three segments of L = 6,717 nucleotides (nt), M = 4,305 nt, and S = 702 nt and were closely related with nucleotide identities of 99.7% to 100%. Phylogenetic analyses based on nucleotide sequences of entire RdRp, glycoprotein and nucleoprotein ORFs showed that the four Kenyan strains shared an MRCA with NRIV Adrar strain isolated from a goat in Mauritania in 2010 (16) (Fig. 3). All NRIV sequences formed a monophyletic clade in sister relationship to BUNV sequences based on L and S segment derived phylogenies and the NRIV M segment sequences were placed as a sister clade to BATV sequences as observed in previous studies.

### Undetected circulation of NRIV in livestock in Kenya.

**FIG 4:**
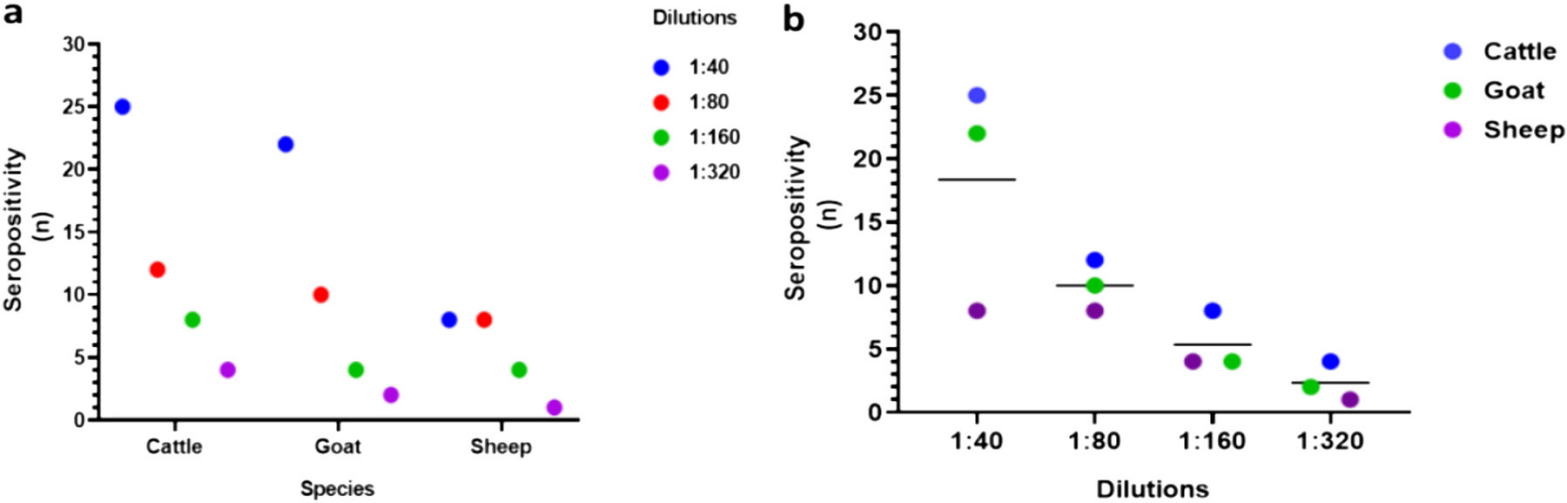
Number of livestock samples positive for NRIV neutralizing antibodies per species (a) and at various PRNT dilutions (b). The n on *y* axis represents the number of individuals per species that were tested positive. The perpendicular line within the graph represents the mean of individuals that were tested positive. The graph was constructed using GraphPad Prism 9.3.1.

To assess the proportion of animals that have been exposed to NRIV, sera from both study sites were tested for presence of antibodies against NRIV. Analyses of 144 sera by indirect immunofluorescence assay (IIFA) identified 60 reactive samples (41.6%; 95% CI = 0.34 to 0.5) of which 55 (38.2%; 95% CI = 30.2 to 46.8) were confirmed by Plaque Reduction Neutralization Test (PRNT) with titers ranging from 1:40 to 1:320 (Fig. 4). NRIV seroprevalence rates in cattle, goats, and sheep were 41.6% (25/60; 95% CI = 30 to 54.3), 52.4% (22/42; 95% CI = 37.7 to 66.6), and 19% (8/42; 95% CI = 9.7 to 33.6), respectively. The proportion was higher in goats and lowest in sheep at both sites (Table 2). NRIV antibody prevalence did not significantly differ between Baringo (33.3%, 24/72) and Kajiado counties (43.1%, 31/72; Fisher exact test odds ratio [OR] 1.4, 95% CI = 0.83 to 2.66; *P* = 0.18).

**TABLE 2:** Summary of NRIV-neutralizing serum samples from cattle, sheep, and goats

Sites				Species^*a*^			Sex^*a*^		Age (yrs)^*a*^				
	Location	Total sample	Total No. Pos	Cattle	Goats	Sheep	Female	Male	3	2	1	Prevalence per location (%)	95% CI
Baringo	Ilngarua	31	10	5 (21)	5 (10)	0 (0)	7 (19)	3 (12)	2 (2)	6 (10)	2 (19)	32.3	18.6 to 49.9
	Perkerra	4	3	3 (4)	0 (0)	0 (0)	3 (4)	0 (1)	1 (1)	2 (3)	0 (0)	75	30.1 to 95.4
	Ngarie	6	3	0 (0)	3 (6)	0 (0)	2 (4)	1 (2)	1 (2)	2 (3)	0 (1)	50	18.8 to 81.2
	Lokuru	5	2	0 (0)	2 (5)	0 (0)	2 (4)	0 (1)	0 (0)	1 (3)	1 (2)	40	11.8 to 76.9
	Sotwa	5	3	3 (5)	0 (0)	0 (0)	2 (4)	1 (1)	0 (0)	2 (4)	1 (1)	60	23.1 to 88.2
	Sintaan	10	0	0 (10)	0 (0)	0 (0)	0 (9)	0 (1)	0 (0)	0 (10)	0 (0)	0	0 to 27.7
	Loropilli	11	3	0 (0)	0 (0)	3 (11)	2 (6)	1 (5)	1 (2)	2 (6)	0 (3)	27.3	9.8 to 56.7
Kajiado	Olesinyai	39	15	14 (30)	0 (0)	1 (9)	14 (33)	1 (6)	3 (8)	9 (23)	3 (8)	38.5	24.9 to 54.1
	Oldorko	16	8	0 (0)	8 (10)	0 (6)	7 (9)	1 (1)	3 (6)	5 (9)	0 (1)	50	28 to 72
	Entasopia	11	4	0 (0)	4 (11)	0 (0)	2 (9)	2 (2)	0 (0)	4 (6)	0 (5)	36.7	15.2 to 64.6
	Birika	6	4	0 (0)	0 (0)	4 (6)	3 (4)	1 (2)	3 (3)	0 (1)	1 (2)	66.7	30 to 90.3
Total		144	55	25 (60)	22 (42)	8 (42)	43 (108)	12 (36)	13 (23)	30 (79)	12 (42)	33.8	

a (), total in different categories.

## DISCUSSION

We report the circulation of NRIV in livestock (cattle, goats, and sheep) from two selected pastoralist ecologies of Kenya. This is the first report of the isolation, detection, and characterization of the virus in livestock hosts in Kenya. Thus far, the virus has been detected sporadically during RVF outbreaks associated with hemorrhagic fever, and this is the first detection apart from an outbreak situation. The virus was first reported in the country during the RVF outbreak in East Africa in 1997 and 1998 in hemorrhagic fever patients from North Eastern Kenya (3, 6, 7, 9, 16). Since then, there has been only one study that reported the presence of NRIV antibodies in febrile patients from different health centers in North Eastern Kenya between 2009 and 2012 (29), but no further report of NRIV infection in humans or livestock has been made, most likely due to lack of specific screening and active surveillance of the virus. However, NRIV has been detected in mosquitoes and ticks from different parts of the country and several vectors were shown to be able to transmit the virus in laboratory experiments (8, 19, 24–28). Thus, depending on the distribution and feeding habits of these vectors, NRIV could be more abundant than available data would suggest. Herein, we found high seroprevalence rates in the three most common livestock species and detected several viremic animals which did not show any obvious signs of disease, a clear indication that the virus is endemic in the country and actively circulating. Importantly, our data indicate the undetected circulation of a zoonotic virus in the three most common livestock species.

There have been few studies carried out testing livestock for NRIV infections, and consequently the epidemiology of NRIV in Kenya as well as in entire Africa remains unclear. As far as to our knowledge, there are only two reports available where NRIV was detected in small ruminants (goats and sheep) during RVF outbreaks in Mauritania in 2010 and 2015 to 2016 (14, 16). Seroprevalence rates below 9% with antibody titers of up to 1:2,560 were detected in small ruminants of varied ages (1 to 10 years) during the Mauritanian RVF outbreak in 2015 to 2016 (14, 16). In contrast, we observed an overall NRIV seroprevalence rate of 38.2%, in small and large ruminants (cattle 41.7%, goats 52%, and sheep 19%) at the age of 1 or 2 years with lower titers of up to 1:320 confirming high exposure of the three livestock species to the virus. The seroprevalence was high in goats in both sites and less in sheep; however, whether this difference is attributed to animal species-specific susceptibility cannot be ascertained due to the small sample size screened and lack of wider geographic scope. There was no significant difference in the seroprevalence rates between the two sites, Baringo county (33.3%) and Kajiado county (43.1%) (Table 2). Of note, none of the NRIV seropositive samples contained antibodies against RVFV or BUNV, which both are endemic in the country. We did not specifically test for the presence of antibodies against BATV, as it is not known to occur in Kenya. Our inference nonetheless is based on a small number of samples, requiring validation of our results through extended surveys employing larger data sets.

We document active circulation of the virus in three presumably healthy livestock species: cattle, goats, and sheep. This pattern is inconsistent with previous detections of NRIV during RVF outbreaks (14, 16). Our detection of NRIV in apparently asymptomatic animals in different areas in Kenya is indicative of a wider geographic circulation that has so far gone unnoticed. The presence of a zoonotic virus in livestock that can easily circulate unnoticed due to mostly asymptomatic or mild infections needs specific monitoring as it can cause disease in humans with severe outcomes. Whether the virus contributes to the disease burden of the livestock population can thus far not be ascertained. Hence, further studies including experimental challenges may be required to understand the potential impact of NRIV on livestock health, as well as to investigate the role of livestock in the epidemiology of NRIV and its possible impact on the associated human population.

Phylogenetic analyses revealed that the NRIV strains detected in this study were genetically most closely related to strains found in small ruminants in Mauritania in 2010 and 2015 (14, 16). Although there is a large geographic distance between the two countries and a relatively long period between the detections in Mauritania in 2010 and 2015 and here in 2020 to 2021, the close relationship could be explained by limited sequence data available for NRIV. This observation could suggest either a low genetic diversity of NRIV, which is supported by the low phylogenetic diversification of the entire NRIV clade, and an expansive geographic circulation in Africa or alternatively a possible exchange of infected animals (or vectors) between the two countries. Interestingly, the available NRIV strains did not cluster according to geographic origin or associated host.

Although NRIV has been isolated from various mosquito species of different genera and a large vector range is suggested, our *in vitro* growth kinetics rather suggest that *Aedes* mosquitoes are more susceptible for an infection with NRIV than mosquitoes of other genera (Fig. 2). However, these cell-culture-based findings need to be further tested using *in vivo* vector competence studies. Several studies detected NRIV in *Aedes* mosquitoes, including the first isolation of NRIV from *Aedes simpsoni* mosquitoes in 1979 in South Eastern Senegal (5, 11, 18).

NRIV has been reported to cocirculate with RVFV in Kenya and with RVF, BATV, and BUNV in Mauritania (6, 7, 14, 16). Factors contributing to the cocirculation are not well understood. However, these viruses infect similar vectors and hosts facilitating simultaneous transmission of multiple viruses between vectors and hosts. The common isolation of BUNV and NRIV from pools of flood water *Aedes* mosquitoes in northern Kenya supports the hypothesis of simultaneous transmission of multiple viruses by mosquitoes (26). The implementation of differential diagnosis for NRIV, RVFV, BATV, and BUNV would be important whenever symptoms of disease compatible with infections with these viruses occur in endemic areas (6, 9, 16, 30). The most recent RVFV outbreak in Kenya was reported in Mandera, Isiolo, Garissa, and Murang’a counties in 2021 (31). It affected animals and humans with a reported case fatality rate of 34% and 55% in humans and livestock, respectively. Surprisingly, only a subset, (20/120) of the samples collected from suspected animal cases was tested positive for RVFV (Central Veterinary Laboratories [CVL] Kabete Lab Report; 31, 32) suggesting the potential contribution of pathogens of unknown etiologies to the outbreak. Moreover, considering the frequent RVFV outbreaks experienced in these areas despite RVFV vaccination campaigns carried out, it would be important to test for the potential contribution of other pathogenic arboviruses known to circulate in the region, such as NRIV, and BUNV, through targeted specific laboratory screening.

### Conclusion.

Until now, NRIV had not been detected aside from RVFV outbreaks. Here, we show NRIV to be actively circulating in apparently asymptomatic cattle, sheep, and goats in two pastoralist-dominated areas in Kenya. Furthermore, animals showed high seroprevalence rates of up to 52.4 suggesting unnoticed virus circulation. There is an urgent need to establish diagnostic tools to investigate the potential health impact of NRIV on livestock and human populations.

## MATERIALS AND METHODS

### Study design and sites.

The study, as part of a bigger project meant to improve understanding of arbovirus transmission networks in Kenya, was conducted as a cross-sectional field- and laboratory-based survey. Sampling was performed twice a year in May/June and September/October between 2020 and 2021 immediately at the end of the rainy seasons (March to May, and August to September) in selected sites of Baringo and Kajiado counties on Kenya’s Rift Valley (Fig. 1). The two ecologies are semiarid inhabited by pastoral communities providing high interactions of humans, livestock, and wildlife. Several arboviruses are endemic to Baringo county, like yellow fever virus (33), RVFV, and the recently discovered phleboviruses Ntepes virus, Perkerra virus, Embossos virus, Bogoria virus, and Kiborgoch virus (34, 35).

### Livestock sampling.

A total of 2,039 apparently healthy/asymptomatic cattle (*n* = 715), goats (*n* = 680), and sheep (*n* = 644) aged 1 to 3 years were sampled from the two study sites by a team, including a registered veterinarian and/or animal health technician. Sampling was done twice a year after the rainy seasons when the vector and arboviral activity were presumably high. Approximately 5 mL whole blood was collected aseptically from the jugular vein of each animal into 10 mL BD Vacutainer blood collection with EDTA. Blood for serum was collected into 10-mL BD Vacutainer blood collection plain vacutainers precoated with serum activator. The samples were processed, aliquoted into cryovials, and appropriately labeled. All samples were transported on dry ice from the field to the Martin Lüscher Emerging Infectious Diseases (ML-EID) Laboratory at icipe for immediate testing and/or storage at −80°C until further screening.

### PCR screening.

Five to seven individual livestock serum samples were pooled depending on the species and site, (100 μL per sample) for RNA extraction. Viral RNA was extracted from 140 μL pooled serum using the QIAamp Viral RNA minikit (Qiagen, Hilden Germany) according to the manufacturer’s protocol. A volume of 50 μL of RNA was obtained and used as a template for cDNA synthesis by Invitrogen SuperScript III Reverse Transcriptase. A 20-μL reaction cDNA was prepared by adding 10 μL extracted RNA to 1 μL Random Hexamers (50 mM), 1.5 μL of Invitrogen RT-PCR Grade water and 0.5 μL Thermo Fisher Scientific dNTP Mix (25 mM) to make a total volume of 13 μL reaction (Mixture 1) and incubated at 65°C for 5 min, then placed on ice for 1 min. Mixture 1 was then added to 7 μL mixture 2, containing 1 μL Invitrogen SuperScript III Reverse Transcriptase, 1 μL Invitrogen RNaseOUT Recombinant RNase Inhibitor, 1 μL Thermo Fisher Scientific USB Dithiothreitol (DTT) and 4 μL 5× RT First Strand buffer, incubated at 15°C for 20 min, 50°C for 60 min, then 85°C for 5 min. The cDNA was stored at −80°C until further use.

Samples were screened by a generic PCR assay using established pan-orthobunyavirus primers targeting the L segment (36). The reaction volume (25 μL) comprised 15.65 μL PCR water, 2.50 μL10×-buffer, 1.25 μL Mg (50 mM), 0.50 μL dNTPs (10 mM), 1.5 μL of 10 μM forward and reverse primers, 0.10 μL Platinum-*Taq* polymerase, and 2.0 μL template (cDNA). Subsequent nested PCR was performed using the PCR product of the first round PCR as template. The PCR conditions were 95°C for 3 min, touch down of 0.5°C per cycle for 10 cycles of 95°C for 15 s, 55°C for 20 s, and 72°C for 40 s, followed by 35 cycles of 95°C for 15 s, 50°C for 20 s, 72°C for 40 s, and a further extension of 72°C for 10 min. The PCR products were electrophoresed in 2% agarose gel stained with ethidium bromide and positive samples purified using ExoSAP-IT PCR product Clean-up Reagent (Applied Biosystems) according to the manufacturer’s instructions, then sequenced in both directions. The sequencing services were outsourced from Macrogen, Europe B.V. Further, RNA was extracted from positive individual serum samples and screened as described.

### Viral isolation.

A confluent monolayer of Vero cells (ATCC CCL-81) grown in 24-well tissue culture plates (Nunc, Roskilde, Denmark) in growth media (GM) containing Gibco Dulbecco’s modified Eagle’s medium (DMEM) enriched with 10% Gibco Fetal Bovine Sera (FBS), 2% Gibco Antibiotic-Antimycotic (100×), and 2% Gibco l-glutamine (200 mM) was used for initial inoculations. Fifty microliters of individual serum samples from NRIV PCR positive pools were inoculated into each well of the confluent monolayer and incubated in a 5% CO_2_ incubator (New Brunswick Galaxy 170 R CO_2_ Incubator Series, Eppendorf, USA) at 37°C for 1 h, rocking after every 15 min. After adsorption, maintenance media (MM) containing 100 μL DMEM supplemented with 5% Gibco fetal bovine sera (FBS), 2% Gibco Antibiotic-Antimycotic (100×), and 2% Gibco l-glutamine (200 mM) was added to each well and incubated at 37°C in a 5% CO_2_ incubator for up to 14 days, observing the cells daily for cytopathic effects (CPE) using Leica DMi1 LED Cell inverted microscope. The positive samples were passaged in a T-25 flask containing confluent Vero cells up to the second passage. The virus was harvested by freeze-thawing the infected cells then centrifuging at 3,000 rpm for 10 min. The supernatant was used for RNA extraction and downstream RT-PCR, next-generation sequencing (NGS), and stored at −80°C until further use.

### *In vitro* viral growth kinetics.

To understand the growth characteristics of the NRIV isolates, *in vitro* growth kinetics were performed in vertebrates cell lines: human, HEK293-T (human embryonic kidney cells); sheep, Llu-L; goat, ZnR (zinc sensing receptor cells); primate, VeroE6 (African green monkey, kidney cells); as well as insects cell lines: U4.4 (Aedes albopictus), CxT (*Culex tarsalis*), Aag2 (Aedes aegypti), AS (Anopheles stephensi), and C6/36 (Aedes albopictus larvae); sand fly, PP-9 (Phlebotomus papatasi). Insect and vertebrate cells were infected with NRIV in duplicates at a multiplicity of infection (MOI) of 0.1 and 0.01, respectively, for RNA extraction and virus titration. Aliquots of infectious cell culture supernatants of vertebrate and insect cells were harvested every 24 h for a period of 6 and 3 days, respectively. Amount of viral genome copy numbers were quantified by real-time RT-PCR using plasmid-based quantification standards. Supernatants were titrated in duplicates on confluent Vero cells in a 24-well plate. The supernatants were diluted in DMEM medium without supplements, cells were washed with 2× PBS, and 200 μL of each dilution was applied on cells. After 1 h of incubation at 37°C and 5% CO^2^, the supernatant was removed, cells were covered with 2.5% Avicel/2× MEM supplemented with 5% FCS and incubated for 3 days at 37°C and 5% CO^2^. Cells were fixed with 6% paraformaldehyde, and the cell layer was stained with crystal violet.

### Genome sequencing and analysis.

The clarified supernatant of isolates from sheep, KE_O93, goat KE_C166 and cattle KE_B02 and KE_B35 were filtered using 0.22-μm filters to concentrate the viral particles and remove any host “contaminants” and bacteria. RNA was extracted from the isolates and libraries prepared using the KAPA HyperPlus kit (Roche, Penzberg, Germany), sequenced using the Illumina MiSeq HTS platform with a designated yield of ~25 million paired-end reads (35, 37, 38).

Raw NGS reads were trimmed, assembled, and analyzed in Biomatters’s Geneious Prime (http://www.geneious.com). The reference-assisted assembly was done using default parameters to obtain a full-length genome sequence of the isolates. The obtained contigs were reconfirmed using BLASTn in the NCBI database (37, 39). Confirmed sequences were translated, and CDS predictions were performed in Geneious Prime. Phylogenetic analysis was performed based on the nucleotide sequences of the L, M, and S segments. Related sequences were downloaded from the GenBank-NCBI database and multiple sequence alignment performed by MAFFT (40). Phylogenetic analysis was performed in Geneious Prime (http://www.geneious.com) with PhyML (41), and a GTR substitution model applying 1,000 bootstrap replicates. To understand the virus evolutionary relationships, the isolated viruses’ closest homologs based on each of the three segments were downloaded from GenBank and used as references in sequence analysis. The inferred phylogenies were visualized in Figtree v1.4.4.

### Indirect immunofluorescence assay.

One-hundred and 44 randomly selected serum samples from cattle (*n* = 60), sheep (*n* = 42), and goats (*n* = 42) were screened for anti-NRIV IgG antibodies using a in house NRIV indirect immunofluorescence assay (IIFA). The slides contained a mixture of KE_O93 NRIV isolate and noninfected Vero E6 cells in a 1:1 ratio fixed on each well except the negative-control well contained noninfected Vero E6 cells. The serum samples were diluted in a ratio of 1:10 with sampling buffer, and 25 μL of the diluted samples applied to the biochip and incubated for 30 min at room temperature. After incubation, the slides were washed twice, 5 min each in wash buffer containing phosphate-buffered saline (PBS), pH 7.2 and 0.2% Tween 20. Twenty-five microliters of Alexa 488 labeled donkey anti-sheep IgG antibodies, donkey anti-goat IgG antibodies, and goat anti-bovine IgG antibodies (Dianova, Hamburg, Germany) for sheep, goat, and cattle, respectively, diluted 1:200 in PBS were applied to each well according to the sample type. The slides were incubated at room temperature in the dark for 30 min, washed twice with wash buffer for 5 min each and then rinsed in distilled water for 2 min. A drop of Prolong Gold Antifade Reagent with DAPI was added, covered with a coverslip, and allowed to dry. Finally, the slides were examined on a fluorescence microscope (Zeiss Fluorescence Microscope).

### Plaque reduction neutralization test.

All NRIV IIFA positive samples were confirmed using plaque reduction neutralization test (PRNT). Vero cells grown in GM were seeded in 24-well plates at a concentration of 1 × 10^6^ cells per well at a volume of 1 mL per well and incubated overnight at 37°C with 5% CO_2_. The cells were observed under the microscope after a day to ensure 70% to 90% confluence and even cell distribution before inoculation. The IIFA positive sera samples were then aliquoted and heat-activated at 56°C in a water bath before serial dilution in maintenance media (MM). Two-fold serial dilutions 1:10 to 1:320 of IFA positive serum samples were prepared in a microtiter plate; then, 30 μL of each serially diluted sera mixed with an equal amount of NRIV KE_O93 isolate diluted to a plaque assay standard concentration that produced 20 to 50 plaques. The mixtures were incubated at 37°C in the presence of 5% CO_2_ for 1 h.

A coincubated mixture of antibody and NRIV virus was used to infect seeded 24-well culture plates containing confluent monolayers of Vero cells after pouring off the growth media and incubated at 37°C for 60 to 90 min in a 5% CO_2_ incubator. After incubation, 2 mL of 2.5% H7509 Sigma-Aldrich methylcellulose (viscosity 4,000 cP) was added to each well and incubated at 37°C in a 5% CO_2_ incubator for 7 days. The plates were then fixed by 3.7% (vol/vol) F8775 Sigma-Aldrich formaldehyde solution prepared in Gibco Dulbecco's PBS pH 7 by pipetting 2 mL per well for a minimum of 2 h and then stained with 0.5% (wt/vol) C0775 Sigma-Aldrich crystal violet prepared in absolute ethanol at 2 mL per well overnight, washed, dried, and were plaques counted manually. PRNT90 positive samples were determined as the reciprocal of the serum dilution giving ≥90% reduction in plaque counts (42).

### Statistical analysis.

The seroprevalence data were analyzed using R version 4.2.0. Comparison of NRIV seroprevalence between the two study sites and different species was done using Fisher exact test. The 95% confidence intervals (CIs) were estimated using the Agresti-Coull method. All tests were performed at a 5% significance level.

### Ethical consideration.

The study was approved by the Kenya Medical Research Institute’s Scientific and Ethics Review Unit (SERU), (SERU No.3312) after gaining approval by the animal care and use committee. Additional approval for the study was accorded by the University of Pretoria, Faculty of Health Science’s Research Ethics Committee (Ethics Reference No. 568/2020).

Sampling of livestock (cattle, goats, and sheep) was conducted by a team, including a Kenya Veterinary Board (KVB) registered veterinarian or/and animal health technician after obtaining written consent from the respective county governments and verbal consent from local farmers.

### Data availability.

The four isolates (KE_C166, KE_O93, KE_B02, and KE_B35) L, M, and S segments sequences were deposited in GenBank under the accession numbers ON755192 to ON755203.
